# Daratumumab efficacy in extramedullary orbital myeloma

**DOI:** 10.1002/ccr3.3458

**Published:** 2020-10-28

**Authors:** Alessandro Gozzetti, Alfonso Cerase, Monica Bocchia

**Affiliations:** ^1^ Hematology Azienda Ospedaliera Universitaria Siena Italy; ^2^ Neuroimaging (Diagnostic and Functional Neuroradiology) Unit Azienda ospedaliero‐universitaria Senese Siena Italy

**Keywords:** daratumumab efficacy in orbital myeloma

## Abstract

Daratumumab is very efficacious in multiple myeloma. Few reports are present about efficacy in extramedullary myeloma. We report here daratumumab efficacy in extramedullary ocular myeloma in a young 46‐year‐old man diagnosed 3 years earlier and relapse refractory to five previous lines of therapy.

## Graphical abstract

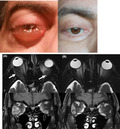

Multiple myeloma (MM) is a monoclonal plasma cell disorder growing within the bone marrow producing a monoclonal protein that can lead to hypercalcemia, renal insufficiency, anemia, or bone disease. Rarely, plasma cells can migrate in the peripheral blood and localize outside the bone marrow giving tumor masses called extramedullary myeloma (EMM).^1^ The incidence is reported to be between 6% and 20%. While MM has seen great survival improvements in the latest years, EMM prognosis is still dismal.^1^, ^2^

A 46‐year‐old man was referred to our division because of a diffuse back pain presented two months earlier. The total skeletal survey showed diffuse osteolytic lesions in his dorsal and lumbar vertebrae and pelvis. Biochemicals showed total protein 10.2 g/dL with gammaglobulin 39% at electrophoresis and a monoclonal component at serum immunofixation IgG/kappa of 3.5 g/dL. Creatinine and calcium were normal. Hemoglobin was 13.5 g/dL. Bone marrow aspirate and biopsy showed a monoclonal plasma cell infiltration of 80%. A diagnosis of multiple myeloma IgG/k stage III A was made. FISH on CD138 selected plasma cells was negative for t (14q) and del 17p. The patient was treated with Velcade‐Thalidomide‐Dexamethasone (VTD) for four cycles and then received autologous stem cell transplantation obtaining complete remission (CR). Maintenance therapy was not allowed at the time. Relapse presented 18 months later with diffuse new osteolytic lesions and serum monoclonal IgG of 3.1 g/dL. He received treatment at first relapse with Lenalidomide 25 mg/day and Dexamethasone 20 mg weekly for 4 months without evident improvement (partial response) and radiotherapy on his right femur. The patient was treated 3 months later at second relapse with Velcade and Bendamustine for three cycles (progressive disease) and subsequently with carfilzomib and pomalidomide 3 cycles (non‐response). After 3 months, he presented to our division with right ocular exophthalmos and chemosis (Figure 1, top left) resistant to oral dexamethasone. Monoclonal IgG was 3.2 g/dL. Bone marrow aspirates showed 60% of monoclonal plasma cells. Orbital magnetic resonance imaging (MRI) (Figure 1, bottom, A) showed disease involvement of right lateral (*closed arrows*) and left inferior (*open arrows)* recti muscles which showed increased thickness, low signal intensity on fat‐suppressed T2‐weighted axial images (A, upper row) and non‐homogenous contrast enhancement on gadolinium‐enhanced fat‐suppressed T1‐weighted coronal images (A, lower row). The patient was treated with anti‐CD38 monoclonal antibody daratumumab (D) monotherapy IV as per schedule. Clinical improvement was seen after 1 cycle of D obtaining a complete clinical resolution (Figure 1, top right). Orbital MRI was repeated 1 month later (Figure 1, bottom, B) and both fat‐suppressed T2‐weighted (B, upper row) and gadolinium‐enhanced fat‐suppressed (B, lower row) images showed a clear cut improvement in muscular thickness resulting in the reduction of bilateral exophthalmos. The patient died for infection with orbital myeloma response 9 months later. Extramedullary myeloma is a very aggressive disease that represents an unmet medical need.^3^, ^4^ D can be efficacious. To our knowledge, this is the first reported case of ocular EMM responding to D therapy.

**FIGURE 1:**
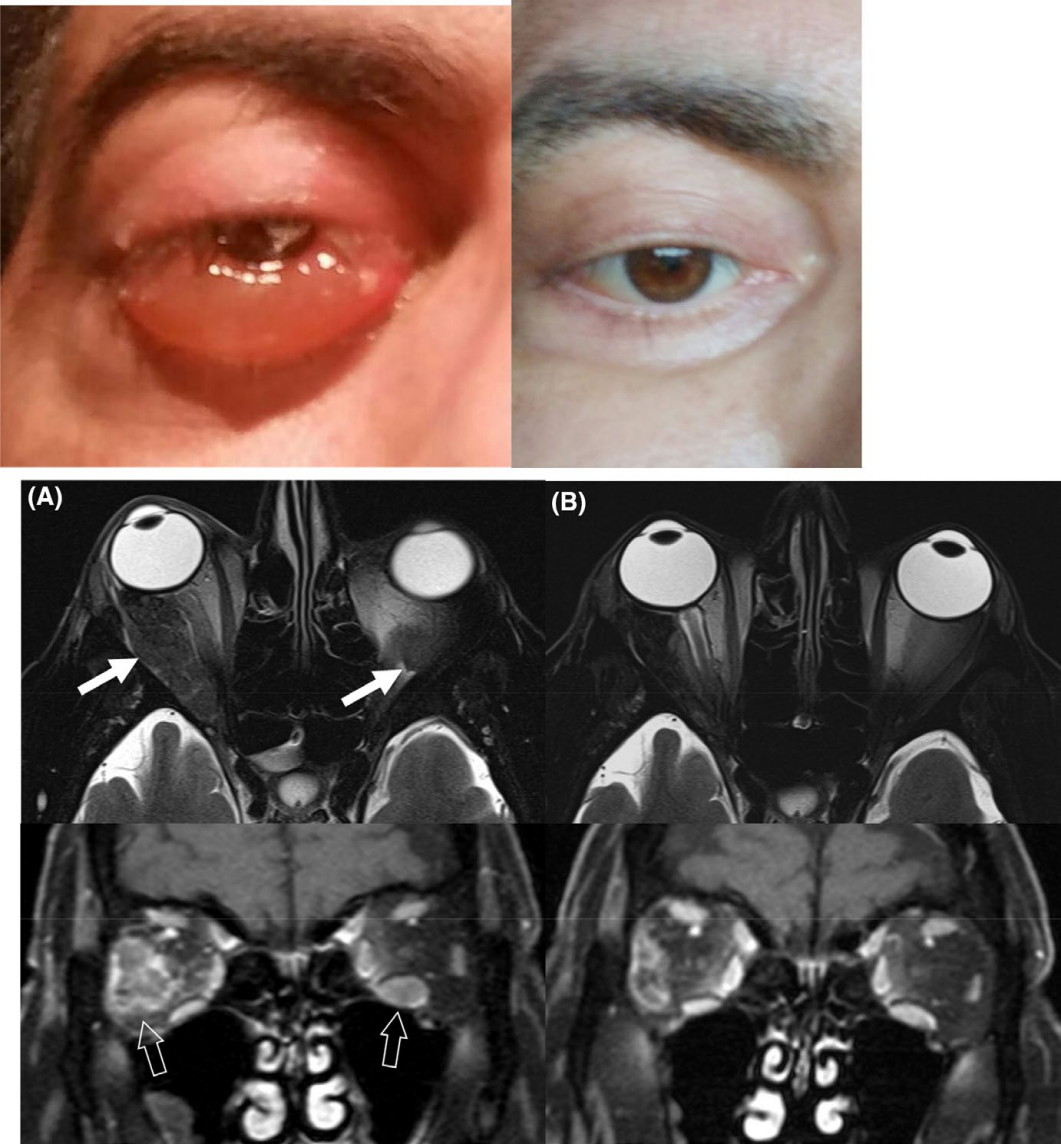
Top left: Patient eye before therapy; A, bottom: orbital magnetic resonance imaging shows disease involvement of right lateral (*closed arrows*) and left inferior (*open arrows)* recti muscles which showed increased thickness; A, upper row and lower row: low signal intensity on fat‐suppressed T2‐weighted axial images and inhomogeneous contrast enhancement on gadolinium‐enhanced fat‐suppressed T1‐weighted coronal images; top right: clinical improvement after therapy; B, bottom, upper row and lower row: orbital MRI 1 mo later fat‐suppressed T2‐weighted and gadolinium‐enhanced fat‐suppressed images, respectively, showed a clearcut improvement in muscular thickness resulting in reduction of bilateral exophthalmos

## CONFLICTS OF INTEREST

The authors declare no conflicts of interests.

## AUTHOR CONTRIBUTIONS

AG: treated the patient and wrote manuscript. AC: performed imaging and wrote the manuscript. MB: supervised and wrote the paper.
